# Comparative analysis of hippocampal extracellular space uncovers widely altered peptidome upon epileptic seizure in urethane-anaesthetized rats

**DOI:** 10.1186/s12987-024-00508-w

**Published:** 2024-01-11

**Authors:** Vanda Tukacs, Dániel Mittli, Éva Hunyadi-Gulyás, Zsuzsanna Darula, Gábor Juhász, József Kardos, Katalin Adrienna Kékesi

**Affiliations:** 1 ELTE NAP Neuroimmunology Research Group, Department of Biochemistry, Institute of Biology, ELTE Eötvös Loránd University, Pázmány Péter Sétány 1/C, Budapest, 1117 Hungary; 2 Laboratory of Proteomics, Institute of Biology, ELTE Eötvös Loránd University, Pázmány Péter Sétány 1/C, Budapest, 1117 Hungary; 3 Laboratory of Proteomics Research, Biological Research Centre, Hungarian Research Network (HUN-REN), Temesvári Körút 62, Szeged, 6726 Hungary; 4 Single Cell Omics Advanced Core Facility, Hungarian Centre of Excellence for Molecular Medicine, Temesvári Körút 62, Szeged, 6726 Hungary; 5 InnoScience Hungary Ltd., Bátori Út 9, Mátranovák, 3142 Hungary; 6 Department of Physiology and Neurobiology, Institute of Biology, ELTE Eötvös Loránd University, Pázmány Péter Sétány 1/C, Budapest, 1117 Hungary

**Keywords:** Microdialysis, Peptidomics, Seizure, 4-Aminopyridine

## Abstract

**Background:**

The brain extracellular fluid (ECF), composed of secreted neurotransmitters, metabolites, peptides, and proteins, may reflect brain processes. Analysis of brain ECF may provide new potential markers for synaptic activity or brain damage and reveal additional information on pathological alterations. Epileptic seizure induction is an acute and harsh intervention in brain functions, and it can activate extra- and intracellular proteases, which implies an altered brain secretome. Thus, we applied a 4-aminopyridine (4-AP) epilepsy model to study the hippocampal ECF peptidome alterations upon treatment in rats.

**Methods:**

We performed in vivo microdialysis in the hippocampus for 3–3 h of control and 4-AP treatment phase in parallel with electrophysiology measurement. Then, we analyzed the microdialysate peptidome of control and treated samples from the same subject by liquid chromatography-coupled tandem mass spectrometry. We analyzed electrophysiological and peptidomic alterations upon epileptic seizure induction by two-tailed, paired *t*-test.

**Results:**

We detected 2540 peptides in microdialysate samples by mass spectrometry analysis; and 866 peptides—derived from 229 proteins—were found in more than half of the samples. In addition, the abundance of 322 peptides significantly altered upon epileptic seizure induction. Several proteins of significantly altered peptides are neuropeptides (Chgb) or have synapse- or brain-related functions such as the regulation of synaptic vesicle cycle (Atp6v1a, Napa), astrocyte morphology (Vim), and glutamate homeostasis (Slc3a2).

**Conclusions:**

We have detected several consequences of epileptic seizures at the peptidomic level, as altered peptide abundances of proteins that regulate epilepsy-related cellular processes. Thus, our results indicate that analyzing brain ECF by in vivo microdialysis and omics techniques is useful for monitoring brain processes, and it can be an alternative method in the discovery and analysis of CNS disease markers besides peripheral fluid analysis.

**Supplementary Information:**

The online version contains supplementary material available at 10.1186/s12987-024-00508-w.

## Background

The brain proteome is highly diverse, and little is known about the protein and peptide composition of the brain extracellular fluid (ECF), which might reflect the cellular processes of the brain. The processing and degradation of macromolecules in cells can be incomplete, resulting in oligopeptides and oligonucleotides beyond amino acids and nucleic acids. Peptides of different sources were identified earlier in the brain secretome [9, 27, 28]. In these studies, the main focus was on secreted bioactive neuropeptides, however, peptides from various precursor proteins were identified as well [9, 27, 28, 71], possibly originating from proteolytic processing and degradation. Indeed, the brain ECF may reflect processes within cells of the central nervous system (CNS) because its composition includes proteins, peptides, neurotransmitters, and metabolites secreted from brain cells.

Clearance of brain ECF is special because the CNS lacks a lymphatic system, and the blood–brain barrier hinders the exit of metabolites, peptides, extracellular vesicles, and degenerated cellular components (debris) from the brain. The cerebrospinal fluid (CSF) was implicated in the clearance of brain ECF by the ECF flowing into the ventricular system [7]. The CSF is produced at the choroid plexus from filtered plasma. Its main protein components account for more than 80% of the total protein content and are the same as in blood plasma [29], which can disturb high-throughput analysis of low abundance, brain-derived proteins. In turn, analysis of CSF has limitations in terms of brain disease diagnosis, which can be investigated via comparison of the peptide composition of tissue ECF and CSF. Due to the difficulties of brain tissue biopsy, peptides secreted by neurons of a diseased brain are a matter of interest in liquid biopsy-based diagnostics.

Changes in ECF of tissues can be analyzed by in vivo microdialysis, which is a direct method for monitoring molecules of a certain size-range determined by the cut-off value of the applied semi-permeable membrane [9, 70]. The dialysis probe consists of an input and an output capillary inserted into a hollow fiber composed of a semi-permeable membrane. The probe is inserted into the tissue, and perfused by artificial cerebrospinal fluid (ACSF) at a slow flow rate. Small molecule compounds, such as neurotransmitters, peptides, and metabolites in the ECF, can enter the hollow fiber by diffusion via ultrafiltration through membrane pores; then, the microdialysate flows into the outflow tubes [47]. Generally, hollow fibers with a cut-off of 5000 Dalton are used for monitoring neurotransmitters and metabolites, however, microdialysis probes made of 50,000 Dalton cut-off fibers allow the detection of larger molecules as peptides. The microdialysate can be analyzed by liquid chromatography-coupled tandem mass spectrometry (LC–MS/MS), and peptides can be identified.

We initiated an animal model study of brain tissue-related peptidome of the ECF to reveal cellular processes reflected in the dialysate. We note that microdialysis can also be performed in human subjects for monitoring extracellularly available molecules [14, 73]. To validate the concept that brain cells release peptides in correlation with changes in cellular processes in physiological and pathological conditions, we selected an epilepsy model for the peptidome analysis.

During epileptic seizures, an increased release of proteins and peptides was proposed [46]. The induction of epileptic seizure is an acute and harsh intervention in brain function, and it activates intracellular and extracellular proteases, which implies an altered brain secretome [40, 66]. Epileptic seizures were induced by 4-aminopyridine (4-AP), a voltage-gated K^+^ channel blocker which is a well-described model for epileptic seizures, and can be monitored by electrophysiology [6, 48], and the *status epilepticus* can be maintained for hours for sample collection. Four-AP prevents the repolarization of neurons, leading to increased neurotransmitter release and epileptic seizures. Epilepsy is a molecularly heterogeneous disease of similar electrophysiological changes. Therefore, the clustering of epilepsy patients by molecular markers could help in stratified treatment. The 4-AP epilepsy model is considered a molecularly homogenous state. We hypothesized that monitoring the ECF peptidome in this model—using in vivo microdialysis—can determine characteristic peptide patterns, and the developed protocol can open the possibility to analyze different types of epilepsies, as well. Our present study revealed a complex, multimolecular peptide change pattern under epileptic activity in rats. Also, we found that some of the altered peptides in the brain ECF are also present in CSF databases. We suggest here that changes in brain peptidome could have diagnostics value after human CSF and serum analysis for verification and translation of peptides abundances in CNS disorders.

## Methods

### Animals

Adult Wistar male rats (weighing 250–320 g) were purchased from Toxi-Coop (Budapest, Hungary). Animals were kept under standard laboratory conditions (lights on at 9:00 AM, lights off at 9:00 PM) in temperature- and humidity-controlled rooms with ad libitum access to food and water. All animal care and experimental procedures were following the Council Directive 86/609/EEC, the Hungarian Act of Animal Care and Experimentation (1998, XXVIII). We carried out experiments in strict compliance with the European Directive 2010/63/EU regarding the care and use of laboratory animals for experimental procedures. All the procedures conformed to the National Institutes of Health guidelines and were in accordance with the ARRIVE guidelines. Animal experiments were approved by the national Scientific Ethics Council for Animal Experiments (PE/EA/01105-6/2022). All efforts were made to minimize the animals’ pain and suffering and to reduce the number of animals used. The animals were randomly assigned to experimental groups, and we assured the experimenters’ blindness to the animal groups whenever possible. A total of 11 rats were used in the experiments.

### Surgical procedure and in vivo microdialysis

Microdialysis probes were made *in house,* as described earlier [30]. Briefly, a hollow fiber (Travenol, cut-off 50,000 Da, diameter of 0.25 mm) was placed into a 25-gauge stainless steel tubing. Fused silica capillaries were used as the inlet and outlet of the probe and were guided by stainless steel tubes.

Male Wistar rats (n = 11) were anaesthetized with 25 (v/w) % urethane (1 ml/200 g) injection intraperitoneally (i.p.) Animals were shaved and placed into a stereotaxic instrument; their body temperature was kept at 37 °C with a heating pad. A medial incision was made on the scalp; then, the cranium was exposed and cleaned with 3% H_2_O_2_. Probes were inserted into the (left and right hemisphere) hippocampus CA3 region (AP: − 4.92 mm, ML: 5 mm, DV: 8 mm) based on the rat brain atlas of [49]. The final position of the probes was attained slowly, in not less than 20 min, in order to minimize the tissue damage. Artificial cerebrospinal fluid (ACSF), containing 140 mEq Na^+^, 3 mEq K^+^, 1.2 mEq Ca^2+^, 2 mEq Mg^2+^, and 144 mEq Cl^−^, was applied for perfusion with a 0.25 µl/min flow rate. ACSF solution was made of amino-acid-free, non-pyrogen water. We started sample collection 1 h after probe insertion to eliminate tissue damage-related artifacts. Control samples were collected for 3 h, then 4 mg/kg 4-aminopyridine (4-AP) dissolved in saline was injected i.p., and samples were collected for 3 more hours after 4-AP treatment. Samples from left and right hemispheres were pooled to yield sufficient sample volume for MS analysis.

### Electroencephalography (EEG) recordings and data analysis

EEG recordings were performed on urethane-anaesthetized rats parallel with the microdialysis sampling. EEG signals were recorded between two stainless steel screw electrodes implanted epidurally above the frontal and occipital cortices. A stainless steel wire was placed between the dorsal neck muscles as the ground electrode. Analog EEG signals were online filtered (0.3 Hz–1.0 kHz) and amplified (gain: 5000) by a 4-channel biological amplifier (BioAmp, Supertech, Pécs, Hungary) and digitized (2.0 kHz, 64 bit) by a CED Micro1401-3 data acquisition device (Cambridge Electronic Design, Cambridge, UK). EEG data collection was performed using the CED Spike2 8.04 software (Cambridge Electronic Design). We analyzed and compared the data recorded just before and 2.5–3.0 h after the 4-AP administration. Two min long epochs were randomly excised from the raw EEG data files and exported to OriginPro 2018 (OriginLab Corporation, Northampton, MA, USA). Signals were then low-pass filtered by an FFT filter at 200 Hz, and power spectra were calculated (Hanning window, 1024 points) using the Signal Processing toolbox of OriginPro 2018. As the 4-AP-induced epileptiform activity resulted in an EEG power increase in the gamma frequency band and in a decrease in the number of urethane-induced slow waves (~ 1 Hz), we calculated the area under the power spectra for ranges 0–5 Hz and 50–80 Hz using the Integrate function of OriginPro 2018. The area data were statistically compared between the control and 4-AP recordings by two-tailed, paired sample *t*-test with a significance level of *P* < 0.05. Before performing the *t*-test, the normality of data distribution was checked by the Shapiro–Wilk normality test.

### Peptidomic analysis of microdialysate samples

Microdialysate samples were purified on Omix C18 tips after acidifying with 1% formic acid. Eluates were dried down, resolved in 0.1% formic acid and subjected to LC-MSMS analysis. Waters MClass nUPLC-Thermo Orbitrap Fusion Lumos Tribrid LC–MS system was used in a data-dependent fashion. Cycle time was 2 s., stepped collision energy was used (30,32,35) for the selected multiple charged peaks up to z = 67. Dynamic exclusion was set to 15 s. Peptides were identified and quantified using the ProteomeDiscoverer (v.:2.4 Thermo) and Byonic search engine [8]. *Rattus norvegicus* sequences of the Uniprot database (02/11/2020, 35848 sequences) concatenated with its reversed sequences was used, without any enzyme specificity. Mass tolerances were set to 5 ppm and 20 ppm for the monoisotopic precursor and fragment masses respectively. Several dynamic modifications were set such as: cleavage of Met and/or acetylation of protein N-termini, oxidation of Met, pyroglutamic acid formation from peptide N-terminal Gln. Relative quantifications were based on precursor intensities.

### Analysis of the number of peptides detected in biological replicates

We analyzed the variation of our results by calculating the number of detected peptides and the distribution of normalized peptide abundances recovered from each probe (see the normalization procedure in the next section). One probe was used for the monitoring of one animal’s control and 4-AP-treated phases. Thus, for each peptide and each probe, we selected the larger abundance value out of control and 4-AP treated phases. Based on the larger abundances, we calculated the number of peptides detected, and visualized the peptide abundances yielded by each probe. We also compared the number of detected peptides before and after treatment. We applied paired, two-tailed Student’s *t*-test to compare the number and mean abundances of detected peptides.

### Statistical analysis of peptidomic data

Peptide abundances were normalized to total peak intensity; then, abundances were normalized again to the mean of peptides with the least variance and abundance ratios. We listed peptides separately that are detected in at least 9 of the 4-AP treated samples and not more than 2 of the control samples upon 4-AP treatment, because peptides that are present only post-treatment may provide relevant markers of 4-AP-induced seizure response.

For statistical analysis, peptides were filtered based on missing values; peptides with more than four missing values in treated or control groups were excluded from further analysis. Mean peptide abundances and coefficient of variation (CV) were calculated for both experimental groups. Spearman correlations were calculated for biological replicates with Python. Peptide cleavage sites were analyzed, and sequence logos were created in R with the ‘ggseqlogo’ package [75].

The normality of data distribution was verified by the Kolmogorov–Smirnov test. Then, paired, two-tailed Student’s *t*-test was applied to peptide abundances from control and 4-AP treatment. Benjamini–Hochberg test with a false discovery rate of 0.25 was performed; we show the adjusted *P*-values in the results. We considered peptides significantly altered with adjusted *P*-value < 0.05 and absolute mean abundance ratio ≥ 2. Hierarchical clustering was carried out using the Euclidean measure to obtain the distance matrix and the complete agglomeration method in R with the ‘heatmap.2’ function of the ‘gplots’ package [77]. The length of detected and significantly altered peptides were calculated and their distributions were compared with two-sample Kolmogorov–Smirnov test in R using the ‘stats’ package [57].

### Dimensionality reduction analysis and feature selection

We performed dimensionality reduction analysis on our dataset to assess important peptides that have a role in the distinction between control and treated samples. For these analyses, missing data were imputed using the Python ‘pandas’ package [43]. Based on peptide abundance data, samples were projected onto a new axis by principal component analysis (PCA) and uniform manifold approximation and projection (UMAP) using Python ‘scikit-learn’ [50] and ‘umap’ [42] packages. PCA is a linear, deterministic reduction technique that transforms large amount of data into principle components. UMAP is non-linear and non-deterministic, it is based on a weighted k-neighbor graph construction, then, a low dimensional layout of this graph is constructed [43].

To know which peptides contribute to the dimensions, we carried out four different feature selection procedures that were applied on omics dataset recently [38], using ‘scikit-learn’, one wrapper, recursive feature elimination (RFE), and three embedded feature selection algorithms. Two of them were random forest-based, namely, random forest classification (RFC) and extra trees classification (ETC); and the third was a regression-based model, lasso. We ranked the feature coefficients of each algorithm. We considered those features important that were among the top ten findings by at least two algorithms.

### Functional annotation and enrichment analysis

Functional annotation and subcellular localization of significantly altered peptides were based on Uniprot (release 2023_01) and Gene Ontology databases ([5, 69]; http://geneontology.org/), respectively. Gene-set enrichment analysis was carried out by g:Profiler (version e106_eg53_p16_65fcd97; [56], https://biit.cs.ut.ee/gprofiler/) g:GOSt online tool with the background of *Rattus norvegicus* genome. For enrichment analysis, accession numbers of proteins that belong to significantly altered peptides were used as input. Terms with less than 4 input proteins and terms with fold enrichment of less than 4 were excluded. Significantly elevated and decreased peptides were run separately. If peptides—belonging to the same protein—increased and decreased, we included them in both runs. Gene-disease association scores were exported from the DisGeNet database using the ‘disgenet2r’ package in R [51].

## Results

### Electrophysiology recordings confirmed epileptic seizures upon 4-AP treatment

**Fig. 1:**
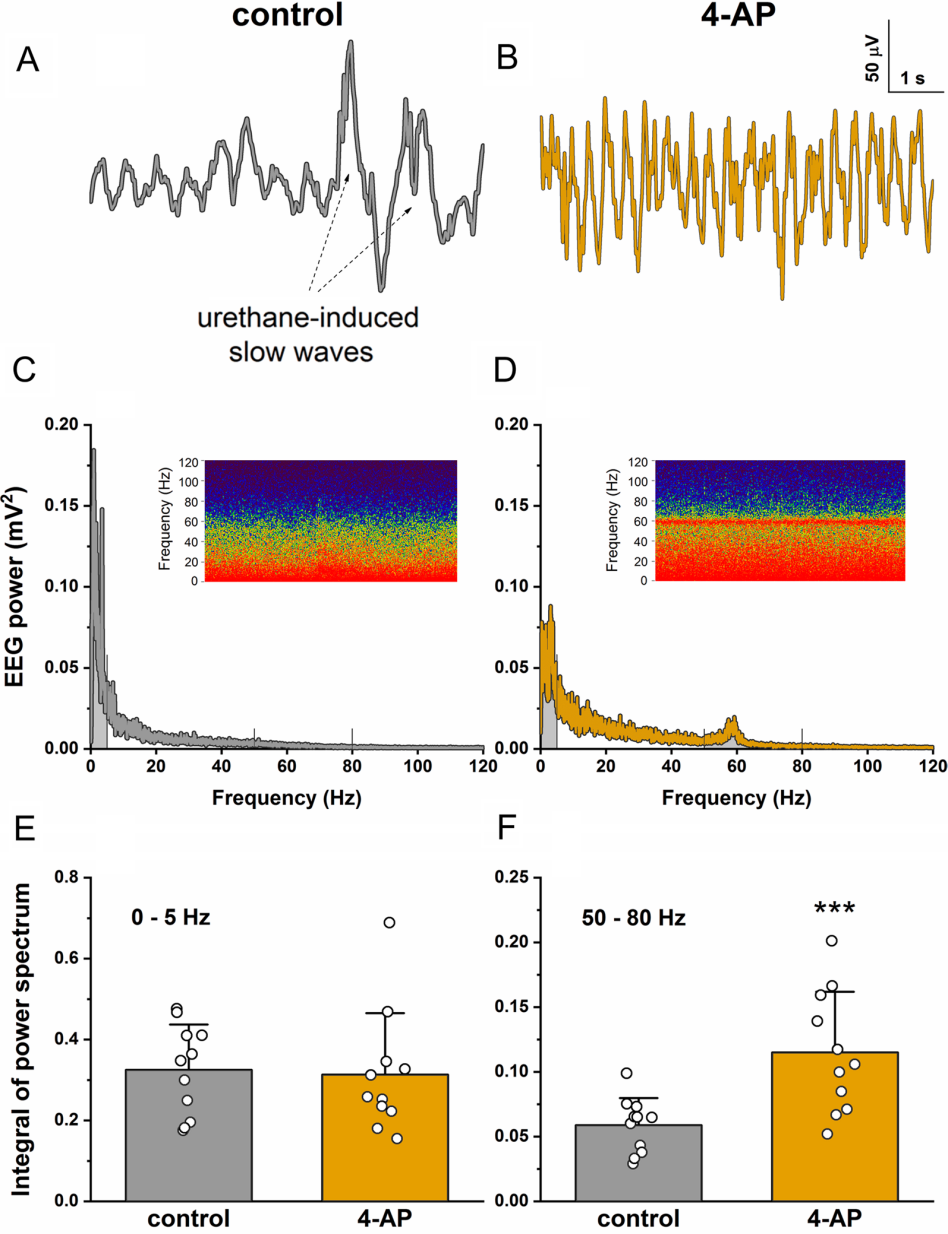
Four-AP treatment induces epileptiform activity in urethane-anaesthetized rats. Representative EEG traces are shown that were recorded between a frontal and an occipital electrode **A** just before and **B** 2.5–3.0 h after 4-AP treatment. The 4-AP-induced activity increase in the higher frequency bands and the abolishment of the urethane-induced slow waves can be seen on the raw traces. **C**, **D** The EEG power spectra and spectrograms (inset figures) show that this 4-AP-induced activity increase was the most remarkable around 60 Hz (i.e., in the gamma frequency range). The spectrograms were calculated from 300 s long recordings and the color scale shows EEG power on a dB scale from 25 to 50 dB. **E**, **F** The area under the power spectra was calculated by integration for ranges 0–5 Hz and 50–80 Hz (noted by lines and shaded areas on plot C and D) and area data were statistically compared using paired sample *t*-test. Data are shown as mean ± SD (n = 11; ***P < 0.001)

The EEG recordings taken before the 4-AP administration were largely dominated by a regular, low-frequency activity (baseline activity), in which high-amplitude slow waves often appeared that are characteristics of urethane anesthesia (Fig. 1A). Contrarily, 4-AP treatment almost completely abolished urethane-induced slow waves and resulted in a regular high-frequency activity with higher amplitudes than the baseline activity in the control recordings (Fig. 1B). These EEG changes could be observed 2.5–3.0 h after the administration of 4-AP. The 4-AP-induced inhibition of cortical slow waves resulted in changes in the low-frequency region of the EEG power spectra (Fig. 1C and D); however, it was not reflected in the area under the curve between 0 and 5 Hz (control: 0.325 ± 0.112, 4-AP: 0.313 ± 0.152, n = 11, *P* = 0.746, paired sample *t*-test, Fig. 1E). At the same time, we found a marked activity increase in the gamma frequency band as shown by a clear peak on the power spectra around 60 Hz (Fig. 1D), similarly to a previously published publication in a mouse study [19]. After integrating the power spectra between 50 and 80 Hz, we found a significant increase in the area under the curve induced by 4-AP treatment (control: 0.059 ± 0.021, 4-AP: 0.115 ± 0.047, n = 11, *P* = 6.33*10^–4^, paired sample *t*-test, Fig. 1F). Thus, our data confirm that 4-AP-induced neuronal depolarization leads to epileptiform activity in cortical networks.

### Peptidomics analysis uncovers widespread peptide changes in microdialysate samples upon epileptic seizure induction

**Fig. 2:**
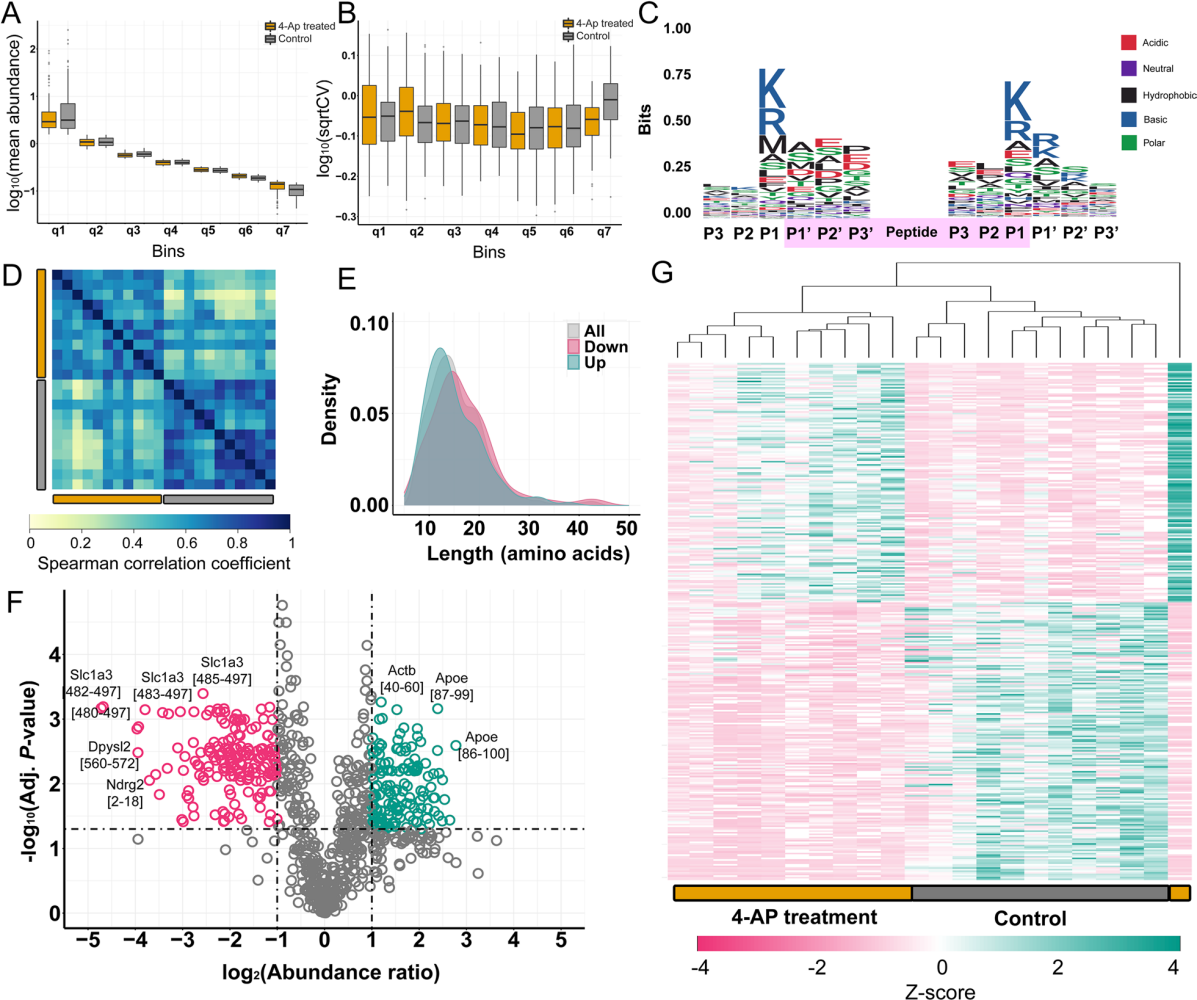
Statistical analysis of peptidome results of 4-AP treated and control samples. **A** Mean abundance distribution of binned data. **B** Square root of coefficient of variation (sqrtCV) distribution shows equal variations from high to low abundance peptides. **C** Sequence logo for all detected peptides show higher frequency for basic amino acids around the cleavage site. **D** Samples show higher correlation coefficients within experimental groups (Spearman’s correlation). **E** Length distribution of all, downregulated and upregulated peptides shows that upregulated peptides are shorter than downregulated one. **F** Volcano plot of detected peptides shows a high number of altered peptides in microdialysate upon 4-AP treatment. **G** Hierarchical clustering separated control and 4-AP treatment groups except for one sample. (n = 11, paired *t*-test; α = 0.05)

We detected 2,540 unique peptides in all the measured microdialysate samples (Additional file 1: Table S1), out of these, 866 were present in more than 6 control and 6 4-AP treated samples. Normalized mean abundances of peptides varied between 0.08 and 166.49 peak intensities. Mean peptide abundances and CV values were uniform between control and treated samples (Fig. 2A and B). Analyzing the amino acid sequences around the peptide cleavage sites, P1 positions were frequently either lysine or arginine suggesting regulated cleavage by proteases with tryptic activity (Fig. 2C). Methionine was also frequent at the N-termini of peptides indicating a large ratio of N-termini fragment of proteins. We also analyzed the ratio of peptides that are the N-terminal fragments of proteins and found high ratio of N-terminal fragments (422; 16.61% of all detected peptides); most of these peptides were acetyl-modified (392; 92.89% of N-terminal fragments; Table 1).

**Table 1:** The number and ratio of N-terminal and acetyl-modified peptides

	N-terminal peptides	All peptides
Peptides without acetyl modifications	30 (7.11%)	2148 (84.57%)
Peptides with acetyl modifications	392 (92.89%)	392 (15.43%)
Sum	422	2540

Replicates were highly correlated with each other within experimental groups, and Spearman correlation coefficients were above 0.5 in all cases (Fig. 2D). To have further insights into the microdialysate peptidome, we have analyzed the length distribution of peptides. Lengths of detected peptides were between 7 and 50 amino acids. Length distributions of significantly down- and upregulated peptides significantly differed from each other (Fig. 2E; Kolmogorov-Smirnoff test: *p*-value = 1.34e−02); downregulated peptides tended to be longer while upregulated ones tended to be shorter with average lengths of 16.79 and 15.06, respectively.

We found high variation of the number of yielded peptides by each probe (1697.27 ± 309.90; mean ± sd), and the distribution of peptide abundance values (Additional file 2: Fig. S1A, B). We compared the number of yielded peptides by each probe before and after treatment. We found that the number of peptides was larger after treatment (Additional file 2: Fig. S1C; paired, Student’s *t*-test, *p* = 0.0346), while the mean abundance of peptides was larger before treatment (Additional file 2: Fig. S1D; paired, Student’s *t*-test, *p* = 0.0128. Thus, more peptides were yielded upon treatment; however, their mean abundance was smaller.

Peptides with significantly altered levels were identified by paired two-tailed Student’s *t*-test followed by Benjamini–Hochberg correction (adjusted *P*-value < 0.05); 322 of which showed at least twofold mean abundance ratio changes upon 4-AP treatment (Additional file 1: Table S2). These peptides were originated from 124 different proteins. Out of the significantly altered peptides, 173 peptides were downregulated and 149 were upregulated. At the same time, peptides that are only detected in 4-AP treated samples could provide high value markers for neuronal damage and hyperexcitability (see Additional file 1: Table S3). For instance, we found Neurogranin (Nrgn) [54–78] in 10 out of 11 4-AP treated samples and only in 1 out of 11 control samples. Amyloid precursor protein (App) [672–681] was detected in all 4-AP treated samples and in 1 of the control samples.

Peptides of fibrinogen alpha (Fga), tubulin alpha-1A chain (Tuba1a), inter alpha-trypsin inhibitor (Itih4), and 4F2 cell-surface antigen heavy chain (Slc3a2) showed the highest increase upon seizures. Levels of several peptides from amino acid transporter Slc1a3 dropped the most (Fig. 2F). Hierarchical clustering of samples could differentiate between control and 4-AP treated groups except for one sample (Fig. 2G).

### Dimensionality reduction algorithms revealed important peptides that distinguish control and treated samples

**Fig. 3:**
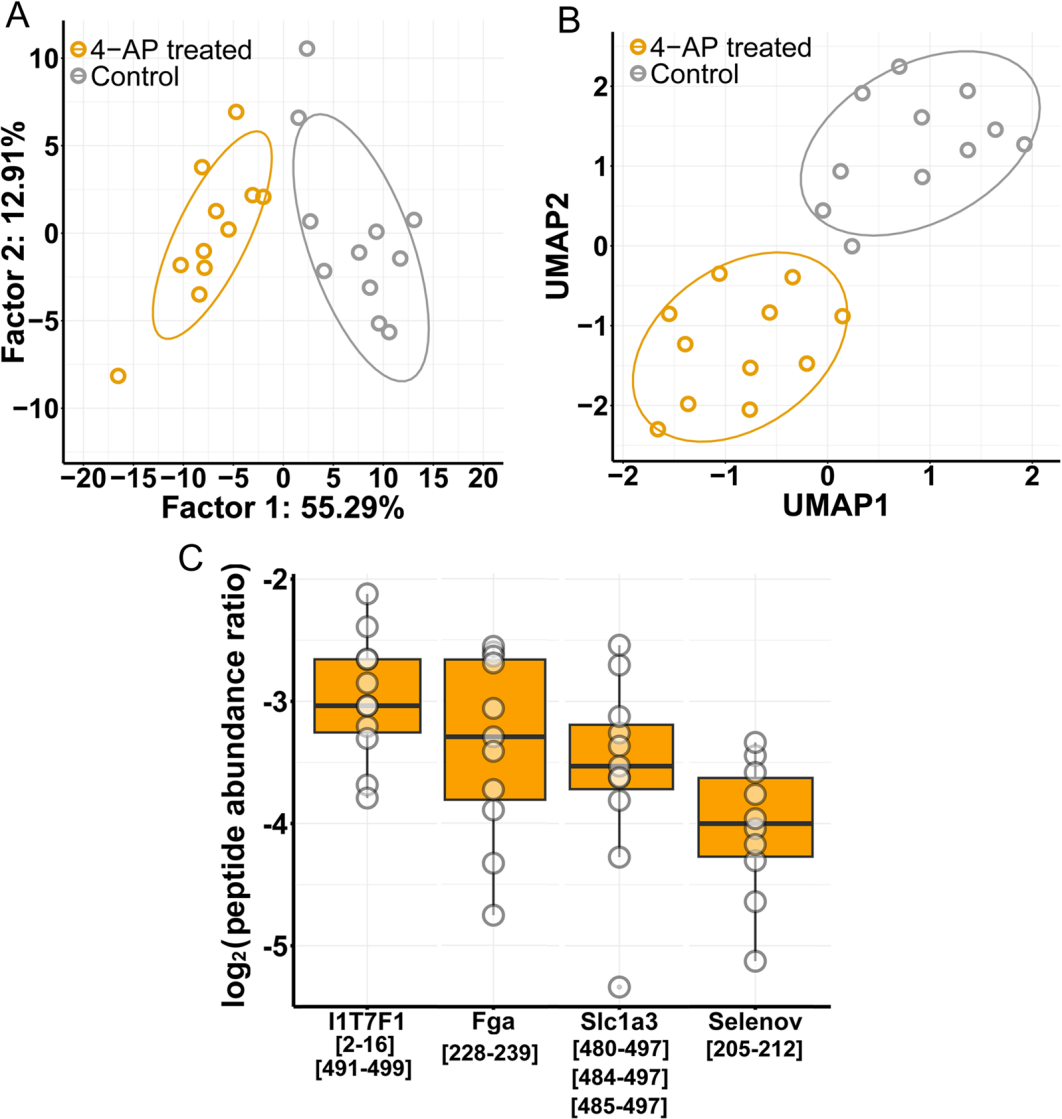
Dimensionality reduction analysis on peptidomics data. **A** Principle component analysis and **B** uniform manifold approximation and projection could separate control and treated samples based on significantly altered peptides. **C** Peptide abundance ratios of important peptides in dimensionality reduction are shown (boxes correspond to 1st and 3rd quartile, median is indicated by line; n = 11). (In cases when several peptides of one protein is shown, the mean of peptide abundance ratios was calculated)

We performed dimensionality reduction analysis on the peptidome changes to assess important peptides—by feature selection algorithms—that have a role in the distinction between control and treated samples. PCA and UMAP separated samples from control and 4-AP treated animals based on the significantly altered peptide abundances (Fig. 3A and B). Interestingly, all important peptides have reduced abundances upon 4-AP treatment (Fig. 3C). Peptides derived from four proteins were selected by at least two feature selection algorithms. Several peptides from the C-terminal of amino acid transporter Slc1a3 were found ([480–497], [484–497], and [485–497]) as an important feature. Peptides from another amino acid transporter were also found, it is a protein without a gene name: I1T7F1. I1T7F1 [2–16] and [491–499] were selected; these peptides correspond to N- and C-terminal of the protein, respectively. Two additional peptides were found: selenoprotein V [205–212] (Selenov) and Fga [228–239]. Hence, amino acid transporters seem to have relevance in the cellular response to seizure induction based on their highly reduced mean abundance and their importance in dimensionality reduction.

### Gene enrichment analysis and functional annotation suggest differentially regulated biological processes

**Fig. 4:**
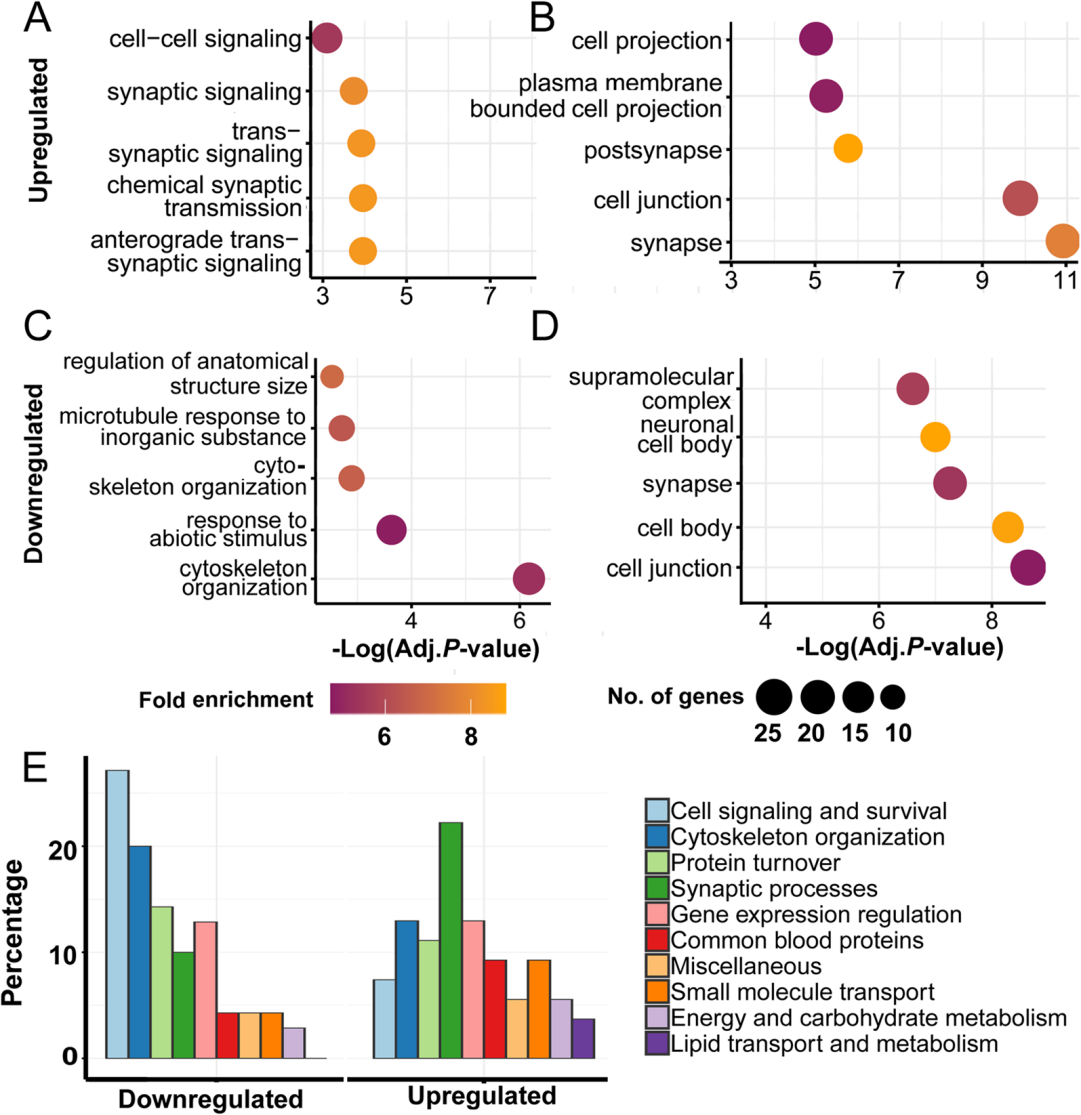
Enrichment analysis of significantly altered microdialysate peptides in response to 4-AP. Enrichment of **A** biological processes and **B** cellular component of upregulated proteins are shown. Enrichment of **C** biological processes and **D** cellular component of downregulated proteins are shown. **E** Manual annotation of protein functions also shows high differences between up- and downregulated peptides. (Enrichment analysis was carried out by g:Profiler (version e106_eg53_p16_65fcd97) g:GOSt online tool with the background of *Rattus norvegicus* genome, and visualized in R)

Enrichment analysis of significantly increased peptides revealed biological processes related to synaptic signaling, such as anterograde trans-synaptic signaling, chemical synaptic transmission, and trans-synaptic signaling (Fig. 4A; Additional file 1: Table S4). In accordance, enriched cellular components included synapse, cell junction, and postsynapse (Fig. 4B; Additional file 1: Table S4). However, decreased peptides were enriched in cytoskeleton process-related terms, such as cytoskeleton organization and microtubule cytoskeleton organization (Fig. 4C and D; Additional file 1: Table S5). Functional annotation of significantly altered peptides also showed distinct patterns dependent on up- or downregulation (Fig. 4E, Additional file 1: Table S6). The most highly represented functional group among elevated proteins was synaptic organization where 22% of proteins belonged (Fig. 4E). In addition, gene expression regulation and cytoskeletal organization had 13–13% of increased proteins. In contrast, cell signaling and survival and cytoskeleton organization were highly represented among decreased proteins with 27.1 and 20%, respectively (Fig. 4E). To sum up, proteins of upregulated and downregulated peptides were mainly enriched in synaptic and cytoskeletal proteins, respectively.

### Peptides of epilepsy-related proteins mostly increased by 4-AP treatment in the microdialysate

**Fig. 5:**
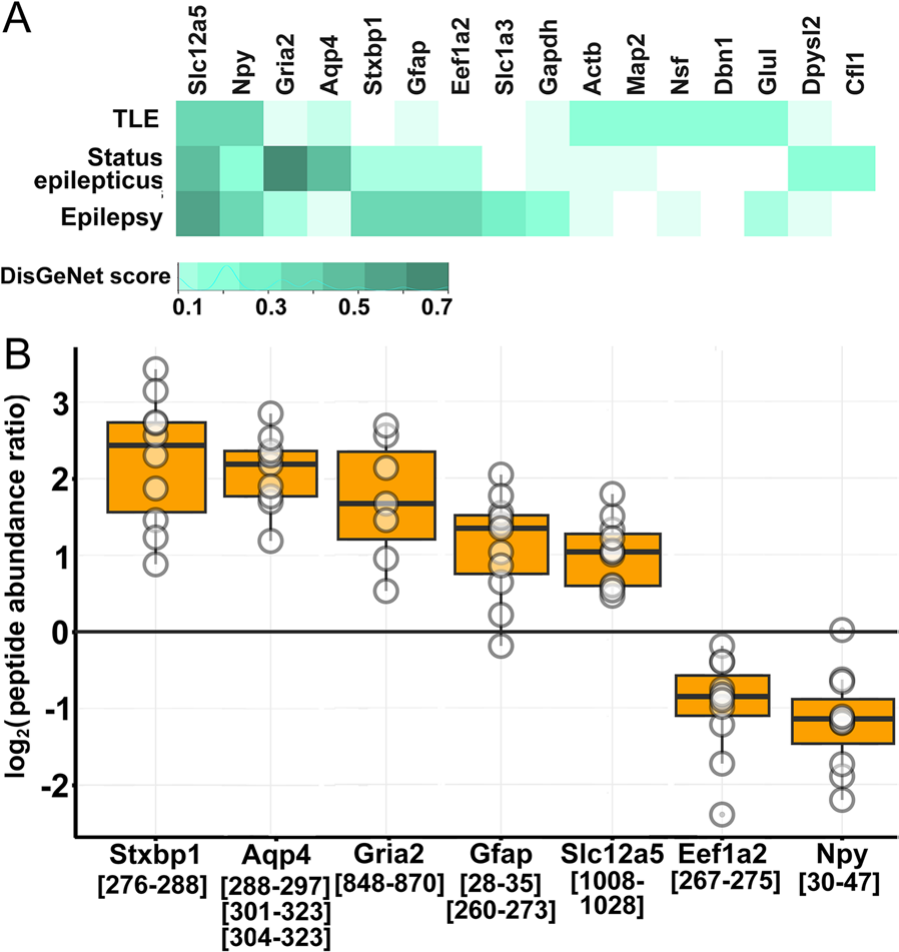
Gene-disease association of significantly altered peptides. **A** DisGeNet scores of proteins that have significantly altered peptides in the microdialysate upon 4-AP treatment are shown in color scale. **B** Peptide abundance ratios of epilepsy-related, altered peptides are shown (boxes correspond to 1st and 3rd quartile, median is indicated by line; n = 11). (In cases when several peptides of one protein is shown, the mean of peptide abundance ratios was calculated)

Our results were validated by the fact that many of the proteins, that had significantly altered peptides, were found to be involved in epilepsy, *status epilepticus*, and temporal lobe epilepsy (TLE) (Fig. 5A) according to the DisGeNet database [51]. Several of them significantly increased (Fig. 5B, Additional file 1: Table S2), such as two peptides from glial fibrillary acidic protein (Gfap) ([28–35]; [260–273]), three peptides from the C-terminal region of aquaporin-4 (Aqp4) ([288–297]; [304–323]; [301–323]), one from glutamate ionotropic receptor AMPA Type subunit 2 (Gria2 or GluA2; [848–870]). Further, we found 1 Aqp4 peptide ([285–300]) that was detected in most of the 4-AP treated samples and in only 2 control samples (Additional file 1: Table S3). Additionally, one peptide from syntaxin-binding protein 1 (Stxbp1; [276–288]), and one from solute carrier family 12 member 5 (Slc12a5; [1008–1028]) significantly increased. Most peptides of epilepsy-related proteins had higher levels upon seizure induction in the microdialysate.

### Neuropeptide fragments decreased upon epileptic seizures in the microdialysate

**Fig. 6:**
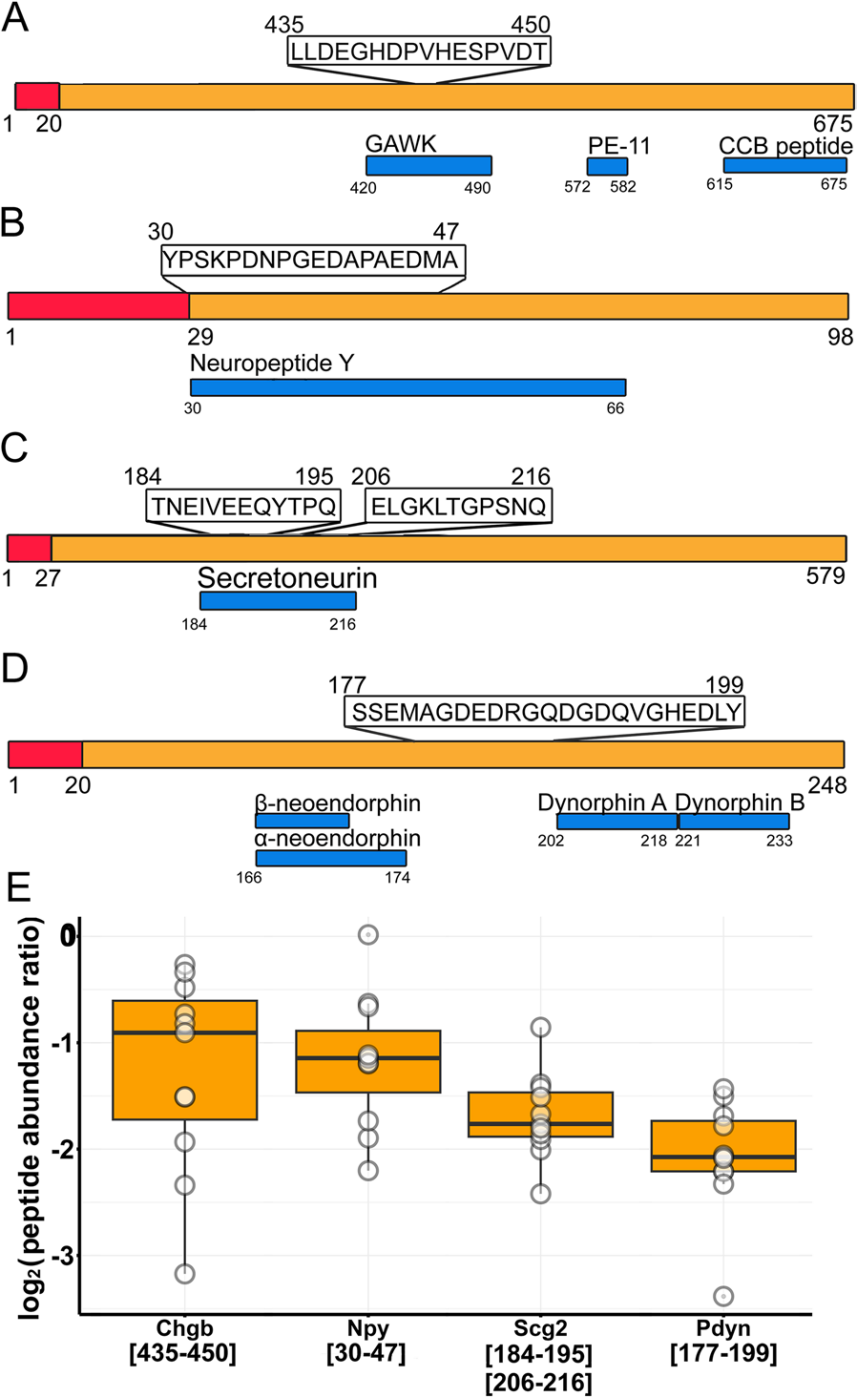
Microdialysate peptides of neuropeptide precursors. Positions of peptides within source proteins **A** Chgb, **B** Npy, **C** Scg2, and **D** Pdyn was illustrated based on Uniprot database. **E** Abundance ratios of significantly altered peptides in the microdialysate (boxes correspond to 1st and 3rd quartile, median is indicated by line; n = 11). (In cases when several peptides of one protein is shown, the mean of peptide abundance ratios was calculated)

Some of the significantly altered peptides of neuropeptide precursors were fragments of biologically active neuropeptides (Fig. 6). Neuropeptide Y (Npy) peptide [30–47] and chromogranin-B (Chgb) peptide [435–450] are also decreased significantly (Fig. 6A, B, Additional file 1: Table S2). Secretogranin-2 (Scg2), of which we found two significantly decreasing peptides ([184–195]; [206–216]) that are derived from its endogen peptide, secretoneurin (Scg2[184–216]) (Fig. 6C, Additional file 1: Table S2). We found a decreasing fragment of proenkephalin-B [177–199] that does not correspond to endogen peptide (Fig. 6D, Additional file 1: Table S2); however it falls right between α-neoendorphin (Pdyn[166–174]) and dynorphyn A (Pdyn[202–218]). In positions 175–176 and 200–201, there is a Lys-Arg amino acid pair which is a common cleavage site for endopeptidases. All significantly altered peptides of neuropeptide precursors decreased in the microdialysate (Fig. 6E).

## Discussion

Here, we analyzed the peptidome changes following 4-AP-induced epileptic seizures in rat brain extracellular space. We detected a very rich extracellular peptidome by applying high molecular weight cutoff dialysis fibers followed by MS-based analysis, and revealed epileptic seizure genesis-induced changes in the brain extracellular peptidome. Our data support the idea that peptides reliably reflect functional processes; several significantly altered peptides were derived from proteins that participate in cellular processes associated with epilepsy models. In the following, we discuss the limitations and the reflected cellular molecular mechanisms of this model and compare to other epileptic seizure models.

### Limitations of the study

Some previous publications confirm that microdialysis can be used for extracellular peptidome analysis [27, 28]. However, it must be taken into account that not only epileptic activity but brain tissue injury enhances the proteolytic activity in the nervous tissue. The insertion of the dialysis probes is an invasive intervention, which might affect our results. BBB damage was found to improve 2 days after probe insertion. Terasaki et al. [68] measured [14C]-sucrose in interstitial fluid (ISF) and plasma to assess BBB damage, and found twofold decline in ISF/plasma ratio 48 h after probe insertion compared to 1 h, indicating that BBB permeability decreases over time. However, 3 days after probe insertion prominent elevation of gliosis markers were observed compared to control; after 1 day, significant elevation was not found [26]). However, a steady-state was reached in 1–2 h after the implantation of microdialysis probes, when extracellular levels of small molecules [68] are measured. Slow probe implantation was associated with improved result quality in the case of acute neuronal recordings measured by silicon probes [20]. Hence, to reduce the potential impact of probe insertion, we implanted the probe slowly, inserted it 8 mm deep over the time course of not less than 20 min, and started the collection of microdialysate samples 1 h after probe insertion. Nevertheless, the BBB is likely impaired upon probe implantation process. We could not avoid the filtration of blood proteins into the extracellular space of the brain; we detected 396 peptides (~ 15.59% of all detected peptides of common plasma proteins [29]. Among the 4 most common blood proteins we detected 3: one albumin peptide [25–48] (albumin accounts for 55% of blood proteins). In addition, we found 273 fibrinogen peptides, 12 peptides from prothrombin, and we did not found any peptides of immunoglobulin. However, we note here that prothrombin [15] expression was reported in the brain, and fibrinogen peptides were found in microdialysate samples before [28].

We detected highly variant responses to 4-AP among animals. Many factors can contribute to the high variation of electrophyisiological and ECF peptidome response of the brain upon 4-AP-induced seizure. These include the variation of i.p. administered 4-AP absorption and brain tissue penetration, electrode and probe implantation, probe permeability etc. The variation of probe permeability is one of the major issues, and that is the reason why we applied self-controlled experimental setting, where we monitor the control and treatment phase in the same animal using the same probe. We found high variation in the number of detected peptides; at the same time, we also found a high number of statistically significant peptides upon treatment, which demonstrates the relevance of the experimental setting. We note here that our results are not quantitative measurements of peptide levels; our results are comparative, meaning they can be interpreted as abundance ratios of peptides in the microdialysates of treated and control phases.

Another major source of variation is the 4-AP absorption and its impact on the brain. To monitor the effect of 4-AP on the brain, we registered electrophysiological recordings, and found significant increase of EEG power around 60 Hz. However, the standard deviation was larger in the treatment phase compared to the control phase, implying large variation in the exposure of brain to 4-AP which could contribute to the large variations found in the peptide abundances as well.

It should be noted that the applied urethane anesthesia reduces epileptiform activity. Al-Gailani et al. investigated the effects of commonly used anesthetics on genetic absence epilepsy in WAG/Rij rats [2]. It has been shown that the administration of ketamine, ketamine/xylazine, urethane, chloral hydrate, pentobarbital, isoflurane, dexmedetomidine, and ddexmedetomidine/ketamine significantly decreased the total number of spike-wave discharges (SWD), the total number of spikes, and the SWD duration.

### Possible sources of peptides in microdialysate

The main source of peptides in the brain tissue is the degradation and processing of proteins by a large-scale of proteases present intracellularly and extracellularly [23, 40]. Theoretically, incomplete protein synthesis can be a source of peptides, however, the biochemical mechanism of protein synthesis makes it implausible. The protein degradation-derived peptides could find binding sites on proteins and remain intracellular and may stabilize the intracellular protein matrix [22]. We speculate that excess peptides are released to the extracellular space and further peptide degradation is performed by extracellular proteases. The abundance and activity of several intra- and extracellular proteases are elevated in epilepsy models [23, 40]. In addition, abundances of polyubiquitinated proteins increased, and the UPS activity was downregulated upon kainate-induced *status epilepticus* [54], implying disturbed protein turnover.

Peptidomics studies of the brain [22] or brain extracellular matrix [9, 27, 28] have implicated that there is a large number of peptides in the brain. Some of them are widely studied bioactive neuropeptides, however, a great ratio of them was not detected before [9, 28]. Furthermore, a large number of intracellular peptides were found in the mouse brain peptidome as well [22]. A fraction of the intracellular peptides may bind the cellular protein matrix and stabilize its 3D structure, however, some others might be released when the cell volume or the cellular protein assembly changes. One of these changes is the general cellular shock-induced compaction of cells [24]. Epileptic seizures result in severe cellular shock of neurons, and the formation of ‘dark’ neurons was observed following 4-AP-induced epileptic seizures [6]. We hypothesize that the alterations of the cellular state, such as ‘dark’ neuron formation, might contribute to peptide release.

The interpretation of peptidome alterations is ambiguous; at the same time, we assume that the increase in peptide abundance may reflect elevated proteolytic activity, increased protein synthesis, increased release from the cells, or reduced ECF clearance. Similarly, the decrease in peptide abundance may reflect reduced proteolysis, decreased protein synthesis, a decrease in release from the cells, or augmented ECF clearance.

In our data, significantly up- and downregulated peptides showed different length distributions. At maximal density, the lengths of upregulated peptides were shorter than the lengths of downregulated peptides. Upon seizures, the increased activity of several intra- and extracellular proteases might result in shorter peptide fragments. In addition, the presence of N-terminal fragments among detected peptides was high; most of these fragments were N-acetylated, which protects peptides from aminopeptidases. A high ratio of terminal fragments could indicate that these peptides are more stable or have higher accessibility for proteolytic cleavage than non-terminal protein regions. At the same time, the dominant presence of Arg and Lys at the C-terminal of the peptides suggests trypsin-like endopeptidase activity.

### Signs of impaired glutamate homeostasis in the microdialysate upon 4-AP treatment

Four-AP treatment elevates extracellular glutamate concentration [48]. Extracellular glutamate levels are dependent on the balance of neuronal and glial glutamate release and uptake. We found significantly altered abundances of peptide fragments of two amino acid transporter in the microdialysate that are relevant in seizures (Slc1a3 and Slc3a2). Slc1a3 encodes excitatory amino acid transporter 1 protein, which is expressed in astrocytes and responsible for glutamate uptake. Slc1a3 protein and mRNA levels were reported to be altered upon drug-induced epileptic seizures in animal models [81]. However, the literature is inconclusive about the direction of Slc1a3 protein and mRNA concentration changes because the studies vary in applied models, epileptogenic compounds, brain areas, time of sample collection after seizures, and quantitative analyses [81]. At the same time, changes in glutamate uptake kinetics indicated a faster clearance rate upon kainate-induced seizure [67]. We detected 8 decreased peptides of Slc1a3 upon 4-AP treatment and several were marked as important features in separating treated and control samples. Slc3a2 is responsible for glutamate release as part of the cysteine/glutamate antiporter complex along with the cysteine/glutamate exchanger (Slc7a11). Extracellular glutamate released by glial cells can induce synchronization of hippocampal neurons [3], which can contribute to hyperexcitability. In human TLE, Slc3a2 mRNA levels increased [85]. We also detected increased levels of two peptides of Slc3a2. In addition, two glutamate synthetase (Glul) peptides increased in microdialysate samples upon epileptic seizures. Glul metabolizes glutamate that was taken up by astrocytes. However, Glul had reduced levels in TLE [72].

Our peptidomics results are partly consistent with gene expressional alterations in TLE regarding glutamate homeostasis. Peptide alterations upon 4-AP treatment indicate that impaired glutamate homeostasis has traces in microdialysate samples of urethane-anaesthetized rats; proteins of glutamate uptake had decreasing peptide levels in our study, while proteins of glutamate release and catabolism had elevated peptide levels.

### Astrocyte morphology and swelling in seizures are implicated in microdialysate peptidome

Astrocytes are essential in maintaining neuronal and synaptic functions. Ultrastructural changes were observed in astrocytes upon seizures. For example, in the pilocarpine model of epilepsy, the number of distal branches of astrocytes was lower after seizures [53]. Vimentin (Vim) and Gfap are intermediate filament proteins expressed by astrocytes in the brain that determine astrocyte morphology. Their expression is increased upon astrogliosis [33]. However, morphological remodeling of astrocytes is relatively slow, it appears 2 to 3 days after injury [58]. At the same time, we detected altered peptide abundances of Vim and Gfap in a 3 h time frame. Two peptides derived from Gfap and 2 peptides from Vim increased, and additional 5 Vim peptide levels dropped significantly.

In 4-AP-induced seizures, astrocyte swelling was observed 2 h post-treatment, which can contribute to impaired metabolite and neurotransmitter clearance from brain ECF [17]. Astrocyte swelling also can reduce ECF volume [76]. A major water channel protein of astrocytes, Aqp4, regulates brain water homeostasis and ECF clearance. Aqp4 is upregulated in the hippocampal sclerotic tissue of patients with TLE [37]. In accordance, we also detected 3 of its increased peptides in microdialysate samples and 1 additional that was detected in 10 out of 11 4-AP treated samples and only in 2 control samples, indicating that its abundance might be below the detection limit in control samples; thus, its increase can be assumed upon treatment.

The gap junction 1 (Gja1) gene encodes connexin 43 (Cx43), which is expressed in astrocytes and creates intercellular hemichannels and gap junctions [84]. It was suggested that the connection of astrocytes might augment the damaged area, and Cx43 level was increased in mesial TLE [21]. In addition, downregulating Cx43 expression after spinal cord injury had protective effects on neurons and astrocytes [10]. We detected the decreased level of a Cx43 C-terminal peptide [374–382], and several other peptides were exclusively detected in control samples, indicating that the abundances of Cx43 peptides in 4-AP samples may be below the detection limit; thus, they are decreased compared to control samples. Cx43 C-terminal fragment was reported to be produced by matrix metalloproteinases and might have biological activity [13]. Monocarboxylate transporter 1 (Slc16a1) is responsible for the transport of compounds, such as lactate, pyruvate, and ketone bodies. Slc16a1 was absent on microvessels and upregulated in astrocytes in methionine sulfoximine and perforant pathway stimulation seizure models [36]. In contrast, we detected a decrease of 3 Slc16a1 C-terminal region-derived peptides.

Water and solute homeostasis is crucial in the brain since the skull restricts the increase of tissue volume. Astrocytes regulate brain water homeostasis and ECF clearance. Our results show that molecular changes in astrocytes can be detected in the brain ECF of urethane-anaesthetized rats.

### Peptides of synapse-related proteins increased in microdialysate

Actin is the major cytoskeletal protein that forms filaments upon polymerization. In the brain, cytoskeleton rearrangement has a large role in synaptic plasticity and cellular morphology in general. We found significantly altered peptides from actin and actin polymerization regulators as well. Cofilin-1 (Cfl1) is an actin-depolymerizing enzyme regulating synaptic plasticity through dendrite morphology. Cfl1 expression decreased in response to spontaneous recurrent seizures in pilocarpine-induced epileptic rats [55]. Cfl1 phosphorylation also decreased 2 h after kainate-induced seizures [82]. We detected one decreasing peptide of Cfl1 ([2–22]). Drebrin (Dbn1) is an actin polymerizing enzyme, and its mRNA and protein levels decreased in the hippocampus of pilocarpine-treated rats after 1 week [60]. We detected 4 decreased peptides of Dbn in the microdialysate samples upon 4-AP-induced seizures. Tropomodulin-2 (Tmod2) blocks actin filament polymerization and depolymerization, its expression increased after kainate-induced seizures [65], and we detected one increasing peptide of Tmod2 in the dialysate. Altered peptides of actin-related proteins were in agreement with the direction of expressional changes reported previously [55, 60, 65] and suggest that both actin polymerization and depolymerization decline upon seizure induction.

Potential neuronal damage markers were suggested previously [61, 78], one of them is the neurofilament light polypeptide (Nefl) [4]. Neurofilaments have roles in synapse formation and enlargement. We detected decreasing peptides of neurofilaments in general except for Nefl, which had 6 increasing peptides upon 4-AP treatment. Nrgn was suggested as a biomarker for synapse degeneration [39], and its CSF level correlated with cognitive decline in Alzheimer’s disease [35]. Nrgn level was elevated in the serum of epileptic seizure patients [32] and traumatic brain injury [59], we also detected an increased level of one of its peptides and its C-terminal peptide ([54–78] was detected mostly in 4-AP treated samples, suggesting its elevation upon seizures.

We also found App [672–681], which corresponds to the N-terminal of amyloid-β 1-40 and 1-42 peptides, in all of the treated samples and only in one control sample. The lack of peptide detection in control samples may suggest that its abundance is below the detection limit or missing from the control samples, which may indicate its higher abundance after 4-AP treatment. We note here that missing values in mass spectrometry data may arise from technical issues as well. Upon kainate-induced seizure decreased neuronal and increased glial App immunoreactivity was observed [63]. Interestingly, patients with late-onset epilepsy of unknown origin had significant prevalence of pathologic CSF amyloid-β 1-42 level [12]. Our data and previous observations suggest that the abundance of App and its peptides can be related to epileptic seizures.

Ion homeostasis of neurons is essential in maintaining synaptic transmission and membrane potential. Sodium/potassium-transporting ATPase subunit alpha-3 (Atp1a3) exports sodium out of the cell and imports potassium into the cell. Four-AP induces extracellular K^+^ accumulation in rat hippocampus [44]. Inactivating Atp1a3 mutation is associated with epileptic seizures since it increases the excitability of neurons [86]. We detected 8 increasing peptides of this protein in the microdialysate. The Slc12a5 gene encodes a potassium-chloride cotransporter that maintains low intracellular chloride level. Its variants are related to several epileptic disorders [31], and we found one increasing peptide of Slc12a5 in the microdialysate. Increased microdialysate levels of peptides from ion transporters might indicate their impaired function, which can contribute to the 4-AP-induced hyperactivity of neurons.

The synaptic vesicle cycle has a major role in neurotransmitter release and synaptic transmission. We detected altered peptide abundances of four synaptic vesicle cycle-related proteins: alpha-soluble NSF attachment protein (Napa), beta-soluble NSF attachment protein (Napb), Stxbp1, and proton-transporting two-sector ATPase (Atp6v1a). These peptides were upregulated upon 4-AP treatment. Napa was downregulated in patients with TLE and pilocarpine-induced seizures 7 days post-treatment, and no changes were found in the acute phase (from 6 h and 3 days) [79]. However, kainate-induced *status epilepticus* increased the number of docked synaptic vesicles 7 days post-treatment [11].

Neuronal hyperexcitability in seizures is governed by impaired synaptic functions [18] or synapse loss [34]. In our study, most of the peptides from synapse-related proteins increased upon seizure induction in urethane-anaesthetized rats. Proteins and proteolytic peptides of synaptic and astroglial markers increased in the CSF, serum, and plasma of patients with acute neurological diseases [59, 61, 78]. It confirms that brain ECF can be a reliable source of disease-related markers.

### Neuropeptide levels decreased in the microdialysate in response to seizures

Neuropeptides are neuromodulators that are stored in large dense vesicles in interneurons and released upon high-frequency firing. Npy has a beneficial effect on epileptic seizures as it is considered an endogenous anticonvulsant. Seizures enhanced Npy expression in various animal models [74]. We detected one significantly decreasing peptide from Npy, which may suggest that decreased degradation contributes to the increase of Npy.

Chromogranin b and secretogranin 2 are neuropeptide precursor proteins upon their cleavage neuroactive peptides are created. Chgb and Scg2 mRNA increase in amygdaloid stimulation-induced seizures after 30 min and 2 h [62], we detected 3 and 10 decreasing peptides of Chgb and Scg2, respectively. In *post mortem* hippocampus of TLE patients, chromogranin b immunoreactivity was elevated [52]. Secretoneurin is a Scg2-derived peptide corresponding to 184–216 amino acids of the protein. An increase in secretoneurin immunostaining was observed in kainate-induced seizure 6 h post-treatment in rat hippocampus [41]. Secretoneurin attracts monocytes, eosinophils, and endothelial cells and acts as an angiogenic agent similar to vascular endothelial growth factor. In contrast, we found 2 peptides that are fragments of secretoneurin: Scg2[184–195] and [206–216], and both of them decreased.

Neurosecretory protein Vgf (Vgf) is a secreted neuronal and neuroendocrine protein, which is processed by proteases, resulting in several neuroactive peptides. Vgf expression was induced 3 h after kainate treatment in rats [64], however, we detected one decreasing peptide of Vgf.

All significantly altered peptides of neuropeptide precursors decreased in the microdialysate. Our results are in contrast with expression and immunostaining studies done on seizure rat models where these neuropeptides increased intracellularly upon seizure induction at various time points post-treatment. In microdialysis, measured peptides can be derived from proteolytically cleaved proteins that do not bind to other proteins. Thus, their levels do not always correspond to the expression rate and intracellular immunoreactivity of proteins. In addition, the decreasing activity of neuropeptide-degrading oligopeptidases was shown in PTZ-induced seizures in the hippocampus at 3 h post-treatment [1]. Thus, we speculate that 4-AP-induced seizures might have a similar inhibitory effect on neuropeptide degradation.

### Brain extracellular fluid components are present in cerebrospinal fluid

The acquisition of molecular information for diagnostic purposes via the collection of body fluid samples—called liquid biopsy—can reach a diagnosis in many cases. However, our knowledge about molecular transport from the brain tissue to blood is unclear, particularly the solute transfer through the CSF and the brain extracellular fluid (ECF). The CSF flows through the cerebral ventricles and the subarachnoid space to its site of reabsorption and can be sampled by lumbar puncture. The CSF was also implicated in the clearance of the brain ECF by the ECF flowing into the ventricular system [7]. Thus, components of the brain ECF secretome could be detected in the CSF. We compared the protein list of detected peptides in this study of brain ECF to a CSF proteome database (https://proteomics.uib.no/csf-pr/; [25]) and found 151 proteins that are common in both datasets. Out of these, 21 and 32 proteins had up- and downregulated peptides in our study, respectively. It reaffirms that brain ECF components can be detected in CSF. Since brain ECF is a more direct source of brain-derived molecules than CSF, its analysis might provide novel, potential biomarkers in brain diseases. This approach might be especially useful in the early phase of chronic progressive neurodegenerative disorders, where the diagnosis is limited due to the low abundance of brain-related molecules in peripheral fluids. We suggest that studying animal models at the peptidomic level, besides the traditional neurotransmitter analysis, by in vivo microdialysis can provide information on the molecular mechanisms and the markers of brain processes in CNS-related diseases.

### Future directions

We hypothesize that ECF peptidome partially originates from the degradation fragments of proteins which may reflect intracellular processes. Similar suggestions have been introduced before [9]; however, further studies are needed to confirm it. The subsequent inhibition of proteases, peptidases, protein turnover, and secretion followed by high-throughput peptidome analysis may help the understanding of the ECF peptidome formation and the mechanism of its changes upon interventions. Inhibition of several proteases and peptidases have been applied in epilepsy models, however their influence on brain ECF peptidome was not analyzed. Interestingly, dipeptidyl peptidase IV inhibitor ameliorated seizures in KA-induced epilepsy [83], and tissue-type plasminogen inhibitor, neuroserpin treatment delayed the progression of seizures and enhanced neuronal survivor in the hippocampus upon KA-induced seizures [80]. Proteasome inhibition also had protective effects on the hippocampus in experimental epilepsy [16]. The consolidation of the functional correlation of ECF peptidome composition and intracellular processes would greatly promote the discovery of novel biomarkers in a wide-range of diseases by in vivo microdialysis.

In vivo microdialysis has been applied in human subjects in areas such as brain tumors [45], traumatic brain injury [73], and stroke [14], however, exploratory biomarker research in humans using invasive techniques, such as in vivo microdialysis, is highly discouraged. For this purpose, a more plausible scientific approach is the use of animal models for direct investigation of ECF by in vivo microdialysis to find potential novel biomarkers or target molecules, which should be followed by human CSF and serum analysis for verification and translation. In conclusion, we imply that brain ECF can be a reliable source of disease-related markers not only at the level of transmitters and metabolites but at the peptidomic level as well.

## Conclusion

Here, we analyzed the peptidomic alterations of hippocampal extracellular space upon epileptic seizure induction in urethane-anaesthetized rats. We found extensive peptidomic changes related to various aspects of epileptic seizures, including impaired glutamate homeostasis, changes in synaptic functions, and astrocyte morphology. By analyzing peptide sequences, we found that upregulated peptides are shorter than downregulated ones, and the abundance of Arg and Lys amino acids at peptide termini indicates cleavage by endopeptidases of trypsin-like activities. Thus, our results indicate that analyzing brain ECF by in vivo microdialysis and omics techniques is useful for monitoring brain processes, and it can be applied as an alternative method for revealing and analyzing CNS disease markers besides peripheral fluid analysis.

### Supplementary Information

**Additional file 1: Table S1.** List of detected peptides in microdialysate samples. Peptide sequences, positions, Uniprot accession numbers, protein names, and normalized abundances are shown. (Norm ab: normalized abundance; 4Ap: 4-AP treated samples; ctrl: control samples). **Table S2.** List of significantly altered peptides upon 4-AP treatment (paired *t*-test, adjusted *P*-value < 0.05). Peptide sequences, positions, Uniprot accession numbers, gene symbols, and protein names are shown. **Table S3.** List of peptides that are detected in at least 9 of the 4-AP treated samples and not more than 2 of the control samples upon 4-AP treatment. Peptide sequences, positions, modifications, Uniprot accession numbers, gene symbols, and normalized abundances (Norm ab) are shown. (4Ap: 4-AP treated samples; ctrl: control samples). **Table S4.** Enrichment analysis on upregulated proteins. (Analysis was carried out by g:Profiler (version e106_eg53_p16_65fcd97) g:GOSt online tool with the background of *Rattus norvegicus* genome). **Table S5.** Enrichment analysis on downregulated proteins. (Analysis was carried out by g:Profiler (version e106_eg53_p16_65fcd97) g:GOSt online tool with the background of *Rattus norvegicus* genome). **Table S6.** Functional categories of proteins that had significantly altered peptides in the microdialysate.

**Additional file 2: Figure S1.** Analysis of the number of peptides detected in biological replicates (A) The number of detected peptides and (B) the distribution of peptide abundances yielded by each probe are shown. The difference between (C) the number (*p* = 0.0346) and (D) the mean normalized abundance of detected peptides in control and 4-AP phase (*p* = 0.0128) are shown (boxes correspond to 1st and 3rd quartile, median is indicated by line; n = 11; paired, *t*-test; α = 0.05).

## Data Availability

Data analyzed during this study are included in this published article [and its Additional information files]. The raw mass spectrometry data files are available from the authors on a reasonable request.

## Abbreviations

4-AP: 4-Aminopyridine
ACSF: Artificial cerebrospinal fluid
CNS: Central nervous system
CSF: Cerebrospinal fluid
ECF: Extracellular fluid
EEG: Electroencephalography
LC–MS/MS: Liquid chromatography-coupled tandem mass spectrometry
PCA: Principle component analysis
UMAP: Uniform manifold approximation and projection
SWD: Spike-wave discharges
TLE: Temporal lobe epilepsy
