# Supplementary material for: Comparative analysis of hippocampal extracellular space uncovers widely altered peptidome upon epileptic seizure in urethane-anaesthetized rats
Source: Fluids Barriers CNS. 2024 Jan 11;21:6. doi: 10.1186/s12987-024-00508-w (PMC10782730; doi:10.1186/s12987-024-00508-w)
Supplement: Supplementary file 2 — Additional file 2: Figure S1. Analysis of the number of peptides detected in biological replicates (A) The number of detected peptides and (B) the distribution of peptide abundances yielded by each probe are shown. The difference between (C) the number (p = 0.0346) and (D) the mean normalized abundance of detected peptides in control and 4-AP phase (p = 0.0128) are shown (boxes correspond to 1st and 3rd quartile, median is indicated by line; n = 11; paired, t-test; α = 0.05). [file 12987_2024_508_MOESM2_ESM.pdf]

Additional information

Comparative analysis of hippocampal extracellular space uncovers widely altered peptidome upon epileptic seizure in urethane-anaesthetized rats

Vanda Tukacs[a,b], Dániel Mittli[a,b], Éva Hunyadi-Gulyás[c], Zsuzsanna Darula[c,d], Gábor Juhász[a,b,e], József Kardos[a], and Katalin Adrienna Kékesi*[,a,b,e,f]

[a]ELTE NAP Neuroimmunology Research Group, Department of Biochemistry, Institute of Biology, ELTE Eötvös Loránd University, Pázmány Péter sétány 1/C, 1117, Budapest, Hungary

[b]Laboratory of Proteomics, Institute of Biology, ELTE Eötvös Loránd University, Pázmány Péter sétány 1/C, 1117, Budapest, Hungary

[c]Laboratory of Proteomics Research, Biological Research Centre, Eötvös Loránd Research Network, Temesvári körút 62, 6726, Szeged, Hungary

[d]Single Cell Omics Advanced Core Facility, Hungarian Centre of Excellence for Molecular Medicine, Temesvári körút 62, 6726, Szeged, Hungary

[e]InnoScience Hungary Ltd., Bátori út 9, 3142, Mátranovák, Hungary

[f]Department of Physiology and Neurobiology, Institute of Biology, ELTE Eötvös Loránd University, Pázmány Péter sétány 1/C, 1117, Budapest, Hungary

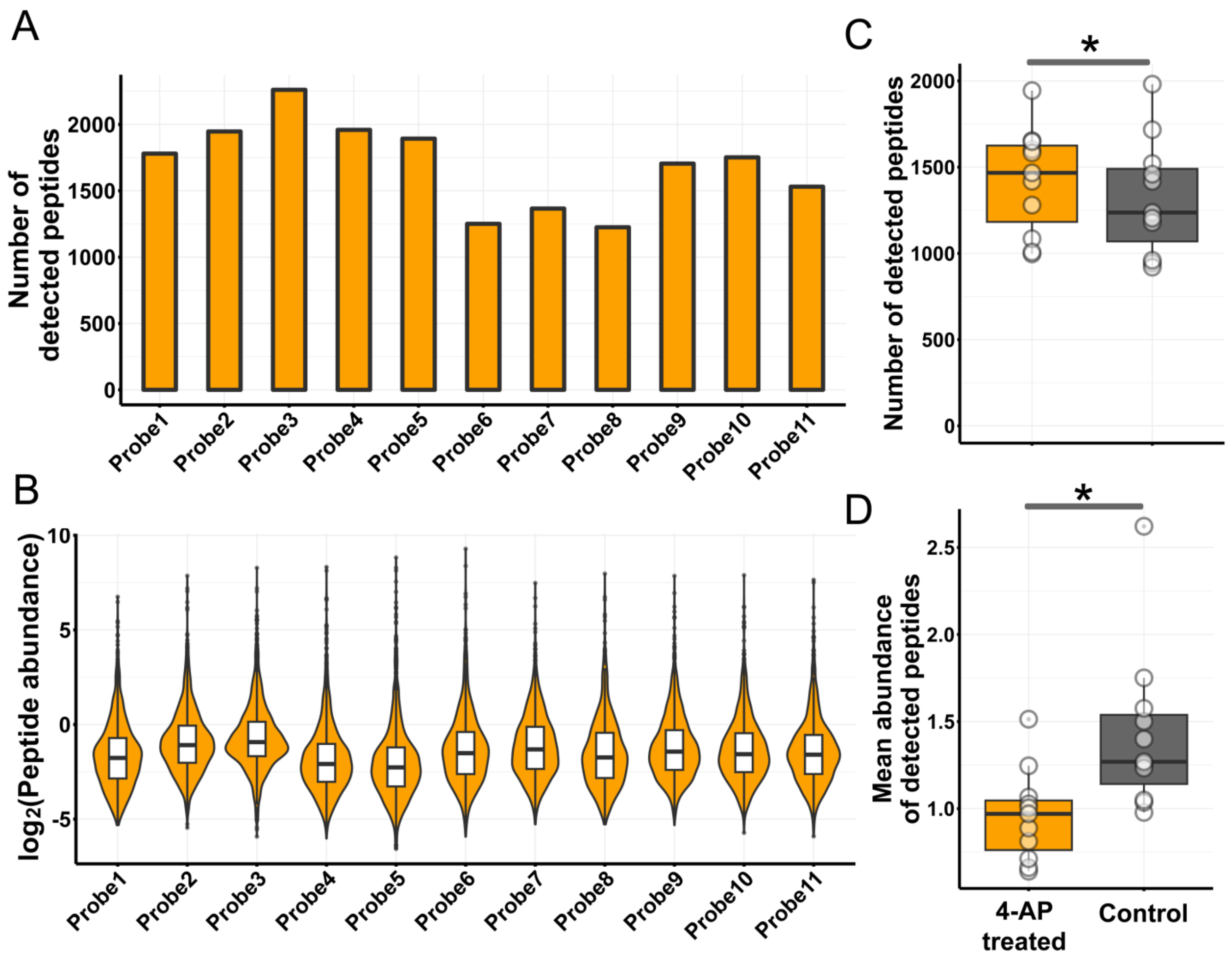

Figure S1 Analysis of the number of peptides detected in biological replicates (A) The number of detected peptides and (B) the distribution of peptide abundances yielded by each probe are shown. The difference between (C) the number ($p = 0.0346$) and (D) the mean normalized abundance of detected peptides in control and 4-AP phase ($p = 0.0128$) are shown (boxes correspond to 1st and 3rd quartile, median is indicated by line; $n = 11$; paired, t-test; $\alpha = 0.05$)
